# The Evolving Role of Immunotherapy in the Treatment with Curative Purpose of Early, Intermediate and Advanced Stage Hepatocellular Carcinoma

**DOI:** 10.3390/curroncol33080492

**Published:** 2026-08-20

**Authors:** Anastasia D. Karampa, Anna Goussia, Evangelos G. Baltagiannis, Nikolaos-Andreas T. Anastasopoulos, Dimitrios K. Christodoulou, Georgios K. Glantzounis

**Affiliations:** 1 HPB Unit, Department of Surgery, Faculty of Medicine, School of Health Sciences, University of Ioannina, 45500 Ioannina, Greece; karampanatasa@gmail.com (A.D.K.); e.bltgs@gmail.com (E.G.B.); n.anastasopoulos@uoi.gr (N.-A.T.A.); 2 Department of Pathology, Faculty of Medicine, School of Health Sciences, University of Ioannina, 45500 Ioannina, Greece; agoussia@uoi.gr; 3 Department of Gastroenterology, Faculty of Medicine, School of Health Sciences, University of Ioannina, 45500 Ioannina, Greece; dchristo@uoi.gr

**Keywords:** hepatocellular carcinoma, immunotherapy, immune checkpoint inhibitors, resectable hepatocellular carcinoma

## Abstract

Hepatocellular carcinoma (HCC) is the most common primary liver cancer and is frequently diagnosed at advanced stages, thereby limiting curative treatment options. Although interventions with curative intent, such as liver resection and ablation, are available, recurrence rates exceed 70%. Recent developments in HCC management have shifted the focus from conventional surgery and targeted therapies to immunotherapy. This review synthesizes current evidence on immunotherapy for curative purposes in HCC, including both neoadjuvant and adjuvant strategies. It evaluates completed and ongoing clinical trials of immune checkpoint inhibitors (ICIs), administered as monotherapy or in combination with anti-angiogenic agents or dual-checkpoint blockade. ICIs may transform the initial treatment paradigm for advanced HCC, although their applications remain in the investigational stage. Preliminary results from the IMbrave050 trial suggested that adjuvant immunotherapy following radical liver resection improves recurrence-free survival (RFS) in high-risk patients. However, the initially promising outcomes of the combination of atezolizumab and bevacizumab compared with active surveillance were not sustained in the median follow-up, and long-term survival results are awaited. Although several studies have demonstrated encouraging preliminary results, a clear survival benefit has not yet been established. Further studies are necessary to establish definitive conclusions. Immunotherapy has a pathophysiological basis and preliminary evidence to redefine HCC management across disease stages; its integration into pre- and post-operative strategies may increase resectability, reduce recurrence, and improve survival. Ongoing trials are expected to clarify its exact role.

## Simple Summary

Hepatocellular carcinoma (HCC) is the most common type of primary liver cancer and is frequently diagnosed at advanced stages, which are generally unsuitable for curative interventions. Among patients who undergo liver resection, approximately 70% experience relapse within five years. Unlike other older therapies, immunotherapy uses Immune Checkpoint Inhibitors (ICIs) to stimulate the body’s immune system to recognize and attack cancer cells. While clinical trials investigating neoadjuvant, perioperative, and adjuvant immunotherapy have demonstrated encouraging preliminary outcomes, the use of these approaches in HCC remains controversial, as they are still considered investigational. This narrative review aims to summarize the current role of immunotherapy in the management of HCC amenable to surgical treatment with curative intent, highlighting recent clinical trial results. Additionally, emerging directions involving novel immunotherapy targets are discussed.

## 1. Introduction

Hepatocellular carcinoma (HCC) ranks as the sixth-most prevalent cancer and is the third leading cause of cancer-related mortality worldwide [1]. The global incidence of hepatocellular carcinoma (HCC) is increasing, mainly due to metabolic-associated steatotic liver disease (MASLD), steatohepatitis, type II diabetes mellitus, and metabolic syndrome. This trend is especially evident in Western countries. Conversely, many Asian countries have seen a decline or stabilization in HCC incidence due to effective hepatitis B vaccination programs and reduced aflatoxin exposure. The epidemiology of HCC is shifting from viral-related to non-viral liver disease risk factors, influencing prevention and treatment strategies [2]. Preventive measures, including hepatitis B vaccination, antiviral treatments for hepatitis B and C, lifestyle changes such as reducing alcohol intake, maintaining a healthy weight through diet and exercise and decreasing exposure to aflatoxins and other hazards, contribute to reducing HCC mortality [3]. Active surveillance enhances outcomes by permitting early detection and curative treatment. However, 50–60% of HCC cases are diagnosed at an advanced stage, largely due to moderate sensitivity and underutilization of surveillance programs. Current management of early- and intermediate-stage HCC involves a multidisciplinary approach, with highly effective curative therapies for early-stage disease and a combination of loco-regional and systemic therapies for intermediate-stage disease [4]. Early-stage management focuses on curative treatments, including liver transplantation, surgical resection, and ablation. Liver transplantation offers the best long-term survival, with 5- and 10-year survival rates of 70% and 50%, respectively [5]. Surgical resection is associated with advantageous outcomes, with a 5-year survival rate of 40–60%. Recent studies indicate a 5-year survival rate of 70% in early-stage patients [6]. According to a retrospective multicenter study, aggressive surgical treatment of HCC, including selected patients at intermediate and advanced stages, provides 34% and 21% five-year survival rates for BCLC B and C stages respectively [7]. However, most HCC cases are diagnosed at an advanced stage. Clinical practice guidelines for HCC are influenced by regional disease etiologies, such as viral hepatitis in Asia and nonalcoholic steatohepatitis (NASH) or alcohol-related liver disease in Western countries.

In the context of immunotherapy for HCC, it is essential to differentiate between recurrence prevention, tumor downstaging, and conversion to surgery. In the management of HCC, recurrence prevention refers to suppressing post-therapeutic disease relapse, particularly micrometastases, following definitive, curative-intent interventions such as primary hepatic resection or ablation. This approach is conceptually distinct from neoadjuvant cytoreduction or maximizing R0 resection margins. In contrast, downstaging specifically involves the use of locoregional or systemic therapies to reduce tumor burden, defined by the size and number of viable nodules, in patients who initially fall outside conventional criteria. This process enables their transition into validated criteria for liver transplantation or curative resection. Conversion therapy, distinct from both recurrence prevention and downstaging, employs systemic and locoregional protocols to downsize advanced-stage HCC or to enhance the future liver remnant. The goal is to transform technically or oncologically unresectable HCC into a surgically resectable disease [8]. Once operability is achieved, complete surgical resection is required for long-term survival and the possibility of cure. According to some studies, conversion therapy can convert 52% of intermediate-stage patients to candidates eligible for resection or transplantation [5].

International societies such as ASCO, AASLD, EASL, and ESMO generally concur on staging systems and foundational treatment principles for HCC. However, notable differences exist in specific therapeutic strategies and the strength of recommendations across various clinical scenarios. A society-specific comparison provides a more nuanced understanding of these distinctions than a generalized Western versus Asian framework.

Regarding neoadjuvant therapy, ASCO, AASLD, EASL, and ESMO do not recommend its routine use outside clinical trials. These guidelines prioritize immediate surgical resection or liver transplantation for eligible early-stage patients with preserved liver function, citing concerns that preoperative therapy may delay definitive treatment and compromise outcomes [9,10,11,12]. In contrast, certain Asian guidelines, including those from the Chinese Society of Clinical Oncology (CSCO), permit consideration of neoadjuvant approaches in selected high-risk or borderline-resectable patients [13]. These approaches may include transarterial chemoembolization (TACE) or hepatic arterial infusion chemotherapy (HAIC), with or without immune checkpoint inhibitors (ICIs).

For adjuvant therapy, ASCO and AASLD have issued conditional recommendations for adjuvant atezolizumab plus bevacizumab in patients at high risk of recurrence following curative-intent therapy, based on emerging evidence from the IMbrave050 trial [9,14]. These recommendations remain provisional pending confirmation of overall survival benefit. EASL and ESMO take a more conservative approach, generally requiring robust phase III survival data before endorsing routine adjuvant systemic therapy and instead favoring surveillance or clinical trial participation [11,12]. Similarly, the Asian Pacific Association for the Study of the Liver (APASL) does not currently support routine adjuvant systemic therapy, although certain regional guidelines, such as those from KLCA-NCC, incorporate specific approaches like cytokine-induced killer (CIK) cell therapy based on local evidence [15].

Regarding downstaging strategies, ASCO and ESMO primarily recognize downstaging as a means to enable liver transplantation, particularly for patients initially beyond standard criteria such as the Milan criteria. AASLD and EASL similarly acknowledge downstaging in transplant pathways but do not broadly apply this concept to surgical resection. Across these guidelines, conversion therapy, defined as treatment intended to render initially unresectable disease resectable, is not formally established as a standard strategy. Instead, systemic or locoregional therapies are preferred for disease control rather than surgical conversion.

In contrast, several Asian guidelines emphasize conversion therapy as a therapeutic goal for patients with intermediate or advanced disease and preserved liver function. These guidelines utilize higher reported response rates from combination regimens, including tyrosine kinase inhibitors, immune checkpoint inhibitors, and hepatic arterial infusion chemotherapy, to facilitate subsequent surgical resection in selected patients [16].

For combinations of immune checkpoint inhibitors with locoregional therapies, ASCO, AASLD, EASL, and ESMO recognize the strong biological rationale and emerging clinical evidence supporting these strategies [14]. However, all societies emphasize that such combinations should currently be restricted to clinical trials or carefully selected cases until more definitive data are available. In summary, while core principles are shared among international guidelines, differences primarily reflect varying thresholds for evidence adoption and regional treatment practices. Table 1 summarizes the differences between Western and Asian guidelines for HCC management.

Historically, combinations of TACE with earlier TKIs did not demonstrate overall survival benefits. However, Western guidelines are now incorporating positive progression-free survival data from recent trials, such as EMERALD-1 (TACE with durvalumab and bevacizumab) and LEAP-012 (TACE with lenvatinib and pembrolizumab), to inform updates to intermediate-stage treatment algorithms [14]. In contrast, Asian guidelines broadly support the upfront use of combination therapies. The Japanese Society of Hepatology and CSCO readily incorporate dual combinations, such as TACE or HAIC with an ICI, employing these modalities earlier in intermediate or advanced stages to address high tumor burdens, prevent TACE refractoriness, and optimize local disease control [15]. Given the positive results of immunotherapy in patients with advanced HCC, researchers are vigorously investigating its role in early- and intermediate-stage HCC.

Ablation is preferred for solitary lesions ≤ 2 cm in diameter in patients with Child–Pugh A, with 5-year survival rates of 70%. For patients diagnosed with HCC and a small (<25%) future liver remnant (FLR), radiological portohepatic vein embolization (RASPE) is a beneficial approach that promotes robust compensatory regeneration of the FLR and enables safe surgical treatment [17,18]. Intermediate-stage HCC has transitioned from palliative care to a stage-migration approach, providing long-term survival (>2.5 years), with TACE as the standard of care.

Furthermore, in advanced stages of HCC, metronomic chemotherapy is a potentially effective and well-tolerated therapy, ensuring in some cases complete remission [19]. The latest 2025 guidelines increasingly recommend using immune checkpoint inhibitors (e.g., Atezolizumab + Bevacizumab) mainly in advanced stages. The advent of ICI-based therapies has recently improved outcomes for patients with advanced-stage HCC. However, median survival remains around 20 months [20], suggesting plenty of space for improvement in therapeutic options. Numerous ongoing trials evaluate clinical advances based on new combination treatments.

The American Society of Clinical Oncology (ASCO) updated its 2020 guideline on systemic therapy for HCC. This latest update was based on evidence from the Check-Mate459 [21] and IMbrave150 [22] studies, as well as 10 randomized controlled trials published through October 2023 that met the inclusion criteria [9]. According to the latest 2025 guidelines, there is no indication for neoadjuvant immunotherapy for patients with early-stage HCC. Currently, there is no defined role for testing microsatellite instability (MSI), mismatch repair (MMR), tumor mutational burden (TMB), or PD-L1 in HCC. The potential of PD-L1 expression at or above 1% as a biomarker for predicting responses to ICIs has not been established in patients with HCC. However, some studies have identified a correlation between transcriptome-based biomarkers and overall survival (OS), with inflamed HCCs appearing to respond well to ICIs. Immune checkpoint inhibition has demonstrated clinical advantages leading to regulatory approvals in patients with HCC [23]. Given the positive results of ICIs in various solid tumor types, such as melanoma and NSCLC, there is significant interest in translating these advances to resectable HCC. Results from one of the first clinical trials of this approach in NSCLC patients were reported in 2018 [24]. The effectiveness and safety of neoadjuvant immune checkpoint inhibitors (ICIs) have been confirmed across various tumor types, with the FDA granting approvals for non-small-cell lung cancer (NSCLC) and triple-negative breast cancer [25]. The success of immunotherapy in treating advanced HCC and the favorable results of perioperative immunotherapy in other cancers have also led to FDA approval [26]. As a result, ICIs may be studied for use as neoadjuvant therapy in resectable HCC [27]. The application of PD-1/PD-L1 antibody therapy as a neoadjuvant treatment has gained interest, with earlier research demonstrating its clinical effectiveness. These therapies have the potential to enhance recurrence-free survival (RFS) rates without causing significant delays in surgical procedures [28]. Ongoing clinical trials are currently investigating the role of perioperative immunotherapy in resectable HCC [29].

Although phase III randomized clinical trials are not yet available, phase II studies have shown promising preliminary results regarding the effectiveness of neoadjuvant ICI treatment for HCC [28,30]. However, as long-term results from these studies are not yet available, caution is warranted. The hypothesis that neoadjuvant–adjuvant ICIs could lead to better clinical outcomes compared to adjuvant ICIs was directly evaluated in the Southwest Oncology Group S1801 clinical trial in patients with stage IIIB to IVC melanoma who were eligible for surgical resection [31]. ICI combination regimens are now established as the standard of care for advanced HCC. Recent phase III clinical trials are investigating ICI-containing regimens in both resectable and embolization-eligible HCC [32].

HCC mainly results from chronic liver inflammation, which leads to a complex tumor microenvironment (TME) composed of immune and stromal cells. Understanding HCC’s TME is challenging as it plays a role in metastasis and tumor resistance. However, given that the TME is a complex system of immune and stromal cells interacting with cancer cells, innovative immune therapies are being developed to target the TME in HCC. Understanding the TME in HCC may provide novel immune therapies to overcome treatment resistance. Tumors can be classified as inflamed or non-inflamed based on the immune microenvironment. Inflamed tumors constitute 30% of HCCs and are characterized by increased expression of immune checkpoints (PD-1 or its ligand PD-L1) and activation of the IFN signaling pathway. Patients with inflamed HCCs are more likely to respond to ICI therapy.

In contrast, HCCs categorized as non-inflamed tend to have a limited response to immunotherapy [33]. Cancer cells adopt various immune evasion strategies, which can affect immunotherapy outcomes. These include increased PD-L1 expression, changes in antigen presentation, and loss of IFNg sensitivity [34]. Immune cells have immune checkpoint receptors that can either promote or inhibit their function. Immune checkpoints promoting T cell activation include CD28, GITR, and OX40, while those with inhibitory action are PD-1, CTLA4, LAG3, TIGIT, and TIM3 [35]. In general, immunotherapy in patients with HCC intends to restore antitumor immunity. This can be achieved with ICIs, monoclonal antibodies that block inhibitory checkpoints or their ligands. Regimens including immune checkpoint inhibitors (ICIs) are now employed as first-line therapies in clinical guidelines [10]. Despite advancements in systemic therapy, similar progress has not been observed in the treatment of early-stage HCC, which includes therapeutic approaches such as resection, liver transplantation, or local ablation [20].

To understand the mechanisms behind the response to immune checkpoint blockade (ICB), researchers analyzed surgically resected tumor lesions and matched healthy adjacent liver segments from patients who were either resistant or non-resistant to neoadjuvant ICB therapy. This study indicated that tumor lesions from patients who responded well were significantly infiltrated by T cells. However, numerous lesions from non-responsive patients also showed a similar enrichment of T cells [36]. By employing multiplex imaging, coupled with single-cell RNA sequencing (scRNA-seq) and single-cell T cell receptor sequencing (scTCRseq) of almost one million liver immune cells, it was discovered that the pathological response to immune checkpoint blockade (ICB) was closely linked to the intratumoral expansion of PD-1hi effector-like CD8+ T cells and CD4+ T cells exhibiting characteristics of T follicular helper cells. scTCR-seq analysis showed that these PD-1hi effector-like CD8+ T cells and dysfunctional CD8+ T cells are two likely outcomes of the proliferative/progenitor-exhausted CD8+ T cell state. Examination of TCR clonal distribution in nearby liver tissue, peripheral blood mononuclear cells (PBMCs), and initial tumor biopsies indicated that numerous T cell clones that proliferated in the tumor following PD-1 blockade were already situated at the tumor site before treatment. According to researchers, CXCL13+ TH and PD-1hi effector CD8+ T cells were enriched in tumor lesions compared with adjacent liver tissue and were expanded to a greater extent in responders than in non-responders [37]. Immune checkpoint blockade provides substantial amounts of viable tumor tissue, enabling comparative analyses between pathological responders and non-responders. More precisely, advanced proteomics, transcriptomics, epigenetics, and analyses of B cell receptor (BCR) and T cell receptor (TCR) repertoires facilitate the examination of individual cells within the treated tumor [38]. Additionally, ctDNA may serve as a valuable marker for precision patient selection, as it reflects tumor burden and micrometastasis, while TLS indicates local immune competence [39]. The combined use of ctDNA and TLS may enable precise identification of patients with resectable HCC at high risk of recurrence who are most likely to benefit from adjuvant targeted immunotherapy [39].

This narrative review provides an overview of current approaches to managing patients with early-, intermediate-, and advanced-stage HCC, with a primary focus on the potential role of ICIs in patients at high risk of recurrence after liver resection, local ablation or liver transplantation as neoadjuvant or adjuvant treatment. Additionally, it lists available evidence from clinical trials of neoadjuvant and adjuvant approaches and discusses ongoing trials. The overall purpose of this narrative review is to gather and evaluate advances and challenges of ICIs in the case of resectable and borderline-resectable HCC. Furthermore, the role of immunotherapy in patients with advanced HCC who can undergo liver resection with curative purpose after successful conversion therapy will be investigated. A range of advanced immunotherapies, including immune checkpoint inhibitors (such as anti-programmed cell death-1 [PD-1], anti-cytotoxic T lymphocyte antigen-4, and anti-PD ligand 1 antibodies), therapeutic cancer vaccines [40], and engineered cytokines, have been utilized in the treatment of HCC.

## 2. Materials and Methods

This is a narrative review with a structured literature search. Major databases, including PubMed, EMBASE, Cochrane Library, and Scopus, were systematically searched for studies published between 2015 and 2026 that evaluated neoadjuvant and adjuvant immunotherapy in resectable, borderline resectable, and advanced-stage HCC for curative purposes. Registered clinical studies were identified from clinicaltrials.gov using search terms such as ‘neoadjuvant immunotherapy,’ ‘adjuvant immunotherapy,’ ‘liver transplantation,’ ‘hepatocellular carcinoma,’ ‘advanced HCC,’ ‘resectable HCC,’ and ‘borderline-resectable HCC.

### 2.1. Inclusion Criteria

(a) Randomized Phase I, II, and Phase III trials; (b) retrospective studies; (c) systematic reviews, (d) meta-analyses, (e) case series and case reports; (f) single-arm trials, (g) non-randomized trials, (h) observational studies, (i) participants aged 18 years or older; (j) treatment regimens including immune checkpoint inhibitors (ICIs) in at least one group; (k) publications in English.

### 2.2. Exclusion Criteria

(a) Editorials; (b) publications in languages other than English; (c) studies lacking relevant data; (d) studies without relevant populations; (e) original literature unavailable; (f) insufficient data for calculation.

## 3. Results

### 3.1. Trials with Neoadjuvant or Perioperative Immunotherapy for HCC Treated with Curative Purpose

Antitumor immune responses depend on the interactions among T cells, antigen-presenting cells, and tumor cells. These interactions are more frequent when a significant tumor burden remains, offering a rationale for the potential superiority of neoadjuvant immunotherapies over adjuvant therapies [41]. Early-phase clinical trials investigating ICIs in HCC, either as monotherapy or in combination, within the neoadjuvant setting, have demonstrated promising preliminary outcomes.

Among published studies on neoadjuvant immunotherapy in hepatocellular carcinoma (HCC), a notable phase I trial evaluated the efficacy of nivolumab combined with cabozantinib, administered over three months, in 15 patients with the objective of curative-intent resection. Twelve patients underwent successful resection, and five achieved a major pathological response, defined as at least 90% tumor necrosis [42]. A single-arm, open-label, phase 2, neoadjuvant trial evaluated the effects of cemiplimab, a high-affinity, fully human immunoglobulin G4 monoclonal antibody targeting PD-1, on treatment outcomes in patients with resectable disease (stages Ib, II, and IIIb). These patients received two doses of cemiplimab and underwent resection 22 days after therapy initiation. Postoperatively, patients received up to eight treatment cycles. Out of 20 patients who underwent resection, 20% had significant tumor necrosis [28]. The limited sample size in this trial necessitates cautious interpretation of the results. However, the pathological responses to cemiplimab observed in this cohort provide a rationale for conducting larger trials to determine the optimal treatment duration and to establish the clinical benefit of preoperative PD-1 blockade in patients with HCC. This trial is registered with ClinicalTrials.gov (NCT03916627, Cohort B) and remains ongoing. The forthcoming results are expected to provide valuable insights. There is considerable research interest in investigating how intratumoral dendritic cell-CD4+ T helper cell niches enable CD8+ T cell differentiation following PD-1 blockade in HCC. According to a neoadjuvant cemiplimab trial, examination of resection samples indicated that tumors responding to treatment exhibited high levels of T cell infiltration. However, some non-responsive HCCs also showed a similar T cell presence [37]. A detailed analysis revealed a significant association between pathological responses to neoadjuvant cemiplimab and the presence of intratumoral triads consisting of regulatory dendritic cells, PD-1- high progenitor CD8+ T cells, and CD4+ T cells displaying characteristics of T follicular helper cells, such as CXCL13 and IL-2. These cellular niches were more commonly found in tumors from patients who responded to the treatment. Nonetheless, the number of patients was small, the study was single-arm, and the clinical value of pathological response as a surrogate for long-term outcomes remains insufficiently validated. According to a randomized Phase II trial, patients received nivolumab, with or without a single dose of ipilimumab, for six weeks before surgery and continued treatment for two years postoperatively. Eligibility required measurable disease, defined as a lesion measurable in one dimension and at least 15 mm by conventional imaging or at least 10 mm by advanced modalities such as MRI or spiral CT. The number of patients enrolled in this study was 30. Significant tumor necrosis was observed in 33% of the nine patients treated with nivolumab alone and in 27% of the eleven patients who received the combination of nivolumab and ipilimumab [43]. Perioperative administration of nivolumab, either as monotherapy or in combination with ipilimumab, appears to be safe for patients with resectable HCC. Although these trials involved small sample sizes, the study of nivolumab combined with ipilimumab demonstrated that ipilimumab was highly effective in patients with cold tumors. This finding supports the hypothesis that CTLA-4 blockade may provide the greatest benefit to patients who lack pre-existing anti-tumor immunity. A multicentre, open-label, randomized phase 2/3 clinical trial involving 294 patients evaluated the efficacy of perioperative camrelizumab combined with rivoceranib versus surgery alone in individuals with resectable HCC at intermediate or high risk of recurrence. At a planned analysis with a median follow-up of 21.3 months, the median event-free survival (EFS) was 42.1 months in the perioperative treatment group and 19.4 months in the surgery-alone group. These findings indicate that perioperative camrelizumab plus rivoceranib significantly improves EFS compared to surgery alone in this patient population [44]. Analysis of data from patients receiving neoadjuvant ICIs will illuminate differences in immunogenicity between HCCs attributed to different risk factors, specifically metabolic steatohepatitis, which has been associated with unique clinical responses. However, as phase III trials have not yet been completed, the use of neoadjuvant immunotherapy warrants caution, given limited long-term outcome data and the risk of adverse effects. Overall, larger trials have to be conducted to explore the utility of applying treatment with ICIs in the neoadjuvant setting, given that profound pathological response can occur early (22 days) before surgery. However, long-term PD-1 blockade is frequently associated with a significant risk of side effects, which may compromise patient compliance. Table 2 summarizes completed clinical trials investigating neoadjuvant immunotherapy in resectable hepatocellular carcinoma (HCC) and their respective endpoints [Refs. [42,43,45,46,47,48,49]].

### 3.2. The Role of Neoadjuvant Immunotherapy in Locally Advanced or Advanced-Stage HCC Patients Who Undergo Liver Resection with Curative Purpose

Recently, clinical trials have aimed to generate robust evidence on the effectiveness and safety of neoadjuvant immunotherapy in patients with locally advanced or advanced-stage HCC. Notably, a recent prospective, single-arm, single-center Phase II clinical trial evaluated the efficacy and safety of a combination of camrelizumab, apatinib mesylate, and mFOLFOX7 regimen (Oxaliplatin, Leucovorin, 5-Fluorouracil) as neoadjuvant therapy for patients with marginally resectable HCC (BCLC stage A/beyond Milan criteria). A total of 14 patients were screened, with 12 ultimately enrolled and administered neoadjuvant therapy. After a median of three treatment cycles, 11 patients underwent liver resection. R0 resection was achieved in all cases, and 7 patients demonstrated a major pathological response (MPR) [50]. The most frequently observed treatment-emergent adverse event (TEAE) was elevated alanine aminotransferase. No serious adverse drug reactions were reported. However, due to the small sample size, definitive conclusions cannot be established.

The literature documents case reports of patients with initially unresectable HCC who underwent preoperative immunotherapy, subsequently enabling surgical resection [51]. In the intermediate stage of HCC, a subset of cases remains unresectable and demonstrates poor response to locoregional treatments. For patients in this group who are unsuitable for or unresponsive to TACE and are candidates for systemic therapies, ICI-based regimens may represent a promising alternative. Recent Phase III trials, such as EMERALD-1 and LEAP-012, have shown that combining ICI-based treatments with TACE can improve PFS [52]. However, when considering adding durvalumab–bevacizumab or pembrolizumab–lenvatinib to TACE for patients with intermediate-stage HCC, the latest guidelines from the European Society for Medical Oncology (ESMO) advise a shared decision-making approach, primarily due to the lack of complete overall survival (OS) data [12]. A recent case report describes a patient with a large HCC who received SBRT and immunotherapy with anti-VEGF therapy. The patient exhibited a complete pathological response, as confirmed by the surgical team following right hemi-hepatectomy, with a fully resected 70 mm necrotic liver mass, absence of residual HCC, and no evidence of vascular invasion [53].

Another case report describes a 54-year-old male patient diagnosed with BCLC-C HCC and a high tumor burden score. The patient initially received apatinib. Following a rupture of the HCC, emergency TACE was performed to control hemorrhage. The treatment regimen was subsequently modified to include both camrelizumab and apatinib. Seventeen months later, the patient underwent a left hepatectomy, and the pathological examination demonstrated complete remission. After surgery, the patient received adjuvant therapy, including TACE and immunotherapy. Four years postoperatively, the patient remains in good performance status with no evidence of recurrence [54]. The number of completed trials investigating neoadjuvant immunotherapy in borderline-resectable HCC remains limited. These studies are primarily Phase II clinical trials with small patient cohorts; therefore, their results should be interpreted with caution [Table 3, refs. [50,55,56]].

### 3.3. Trials with Adjuvant Immunotherapy for HCC Treated with Curative Purpose

The prevention of recurrence following hepatic resection or local ablation through adjuvant therapies for HCC has remained a challenging objective for many years. Two systematic reviews have summarized multiple randomized controlled trials assessing the impact of adjuvant therapies on RFS after potentially curative interventions. The most recent analyses included data from seven trials with results published between 2002 and 2020 [33]. The majority of these studies did not demonstrate any significant clinical benefit.

Recent studies suggest that adjuvant therapy with atezolizumab and bevacizumab may improve RFS in patients with HCC following surgical resection or ablation. However, the OS benefit has not yet been established, ICI application remains investigational, and uncertainties remain regarding long-term toxicity and cost-effectiveness. More specifically, the IMbrave 050 trial initially demonstrated improvements in RFS and PFS among patients at high risk of recurrence following liver resection or local ablation. Patients in the intervention group received a combination of atezolizumab and bevacizumab, while those in the control group underwent active surveillance only [57]. Although risk factors for HCC recurrence following liver resection have been thoroughly identified, including a solitary tumor larger than 10 cm in diameter, multinodular disease, elevated serum alpha-fetoprotein (AFP) levels, microvascular invasion, and poor differentiation [58], the IMbrave 050 trial employed expanded criteria to define recurrence risk. Patients were classified as high risk following liver resection if they exhibited any of the following: up to three tumor lesions with the largest tumor exceeding 5 cm; four or more tumors with the largest tumor measuring 5 cm or less; or up to three tumors with the largest tumor 5 cm or less, accompanied by vascular invasion or poor tumor differentiation. A total of 668 patients were randomly assigned to receive either atezolizumab plus bevacizumab (n = 334) or active surveillance (n = 334). After a median follow-up of 17.4 months, the median recurrence-free survival (RFS) was not reached in either group. However, statistical analysis demonstrated a significant benefit for the intervention group, with a longer estimated time to recurrence (*p* = 0.003) [57]. The treatment lasted 11.1 months for atezolizumab and 11.0 months for bevacizumab. The incidence of grade 3–4 adverse events was 41% in the intervention group and 13% under active surveillance.

Additionally, 8.7% of patients discontinued both atezolizumab and bevacizumab due to adverse events. According to the updated data from the IMbrave 050 study, the initial promising results for the RFS with the combination of atezolizumab and bevacizumab compared to active surveillance were not maintained. Additionally, the OS data are still considered immature, and the clinical value of this approach warrants further investigation. Furthermore, the safety profile of the combination treatment remained consistent with that of each agent [59]. Consequently, the results of the IMbrave050 study do not support the use of a combination of atezolizumab and bevacizumab in the adjuvant setting. However, in-depth subgroup analyses indicate potential benefits for specific patient groups, warranting further prospective evaluation.

A phase III randomized clinical trial conducted in Korea involving 230 patients who underwent curative treatment for HCC, including liver resection or RFA, demonstrated that patients receiving cytokine-induced killer (CIK) cell immunotherapy achieved a median RFS of 44.0 months, compared to 30.0 months in the control group. The immunotherapy significantly prolonged both OS and cancer-specific survival. However, a higher proportion of patients in the immunotherapy group experienced adverse events (62% versus 41%) [60].

Lee et al. evaluated the efficacy of activated cytokine-induced killer (CIK) cell injections, comprising CD3+/CD56+ and CD3+/CD56− T cells as well as CD3−/CD56+ natural killer cells, in improving RFS among patients following curative treatment for HCC. The study enrolled 230 patients with HCC who underwent surgical resection, RFA, or percutaneous ethanol injection at hospitals in Korea. Participants were assigned to either an immunotherapy group receiving autologous CIK cells or a control group under active surveillance. The median RFS was 44 months in the immunotherapy group, compared to 30 months in the control group [60]. A recent systematic review demonstrates that adjuvant ICIs, administered as monotherapy or in combination with TKIs or anti-angiogenic agents, improve survival in patients following resection or ablation for HCC. RFS increased significantly across all ICI-based regimens, with no significant difference observed between monotherapy and combination therapies. Subgroup analyses revealed consistent benefits regardless of the curative treatment modality, the use of TACE, or the study design.

Furthermore, OS was superior with ICI-based adjuvant therapies compared to surveillance [61]. By integrating data from trials with mechanistic investigations, it is proposed that the optimal target population for adjuvant targeted immunotherapy should simultaneously fulfill the following criteria: (1) exhibit high-risk clinical features, such as exceeding the up-to-7 criteria, microvascular or macrovascular invasion, and multinodular tumors, (2) demonstrate positive biomarker status, specifically tertiary lymphoid structures (TLS) positivity and postoperative ctDNA positivity and (3) follow a perioperative treatment paradigm that includes a neoadjuvant component [62]. Discrepant findings across recent key trials of adjuvant therapy for post-resection HCC can be attributed to variations in baseline risk severity among enrolled populations. In negative RCTs such as STORM, IMbrave050, and KEYNOTE-937, participants generally exhibited fewer high-risk features and earlier disease stages, which limited the potential for adjuvant therapeutic benefit. In contrast, an analysis of 2787 patients with HCC (961 receiving adjuvant PD-1 inhibitors and 1826 undergoing active surveillance) who underwent curative resection at 17 medical centers in the GUIDANCE multicenter database (January 2019 to January 2025) included cohorts with prominent high-risk features, such as a higher proportion of multinodular tumors and macrovascular invasion. In this population, adjuvant therapy was associated with a significantly longer RFS (31.8 vs. 22.9 months), with similar results observed in the propensity score-matched cohort (32.7 vs. 21.3 months) [62]. Consistent with the adjuvant arm of IMbrave050, the cohort’s recurrence hazard rate peaked at 24 months. The CARES-009 trial, a positive RCT, also enrolled a high-risk cohort characterized by a high proportion of tumors larger than 5 cm, BCLC stage B (31% vs. 29%), and macrovascular invasion, and included a neoadjuvant treatment phase, followed by surgical resection and at least 6 cycles of adjuvant therapy. The combination arm achieved a median RFS of 42.1 months compared to 19.4 months in the active surveillance group [44]. Additional studies focusing on patients at high risk of recurrence, including those with Eastern Cooperative Oncology Group performance status 1 and microvascular invasion, have also demonstrated favorable tolerability and clinical efficacy [63]. These findings suggest that the benefit of adjuvant targeted immunotherapy for post-resection HCC is positively correlated with baseline recurrence risk, and that meaningful therapeutic benefit is likely confined to patients at high risk of recurrence.

Most adjuvant therapies for HCC have not demonstrated efficacy, except for autologous cytokine-induced killer (CIK) therapy, which has been shown to prolong RFS in both RCTs and real-world data (RWD). The present study evaluated the long-term outcomes of adjuvant CIK therapy through an extended follow-up of the original RCT and a retrospective cohort study. The extended RCT follow-up included 226 patients (114 in the CIK group and 112 in the control group), with the follow-up period extended from 2 to 9 years. Simultaneously, a retrospective RWD study was conducted involving 577 patients from two tertiary centers in Korea, comprising 251 patients who received adjuvant CIK therapy and 326 controls. In the RWD study, the CIK group demonstrated significantly prolonged RFS compared to controls both before propensity score matching (median: 101.2 versus 64.7 months) and after matching (median: 101.2 versus 65.7 months). In the extended RCT follow-up, the CIK group exhibited a significantly longer RFS (median: 44.0 versus 30.0 months) compared with the control group [63]. Adjuvant CIK cell therapy significantly improved RFS in both the RWD study and the 9-year extended RCT follow-up, supporting its reproducible benefit in reducing recurrence after curative treatment of HCC. These consistent findings provide robust evidence for the clinical utility of CIK therapy as a durable adjuvant immunotherapeutic strategy for HCC. All completed trials of adjuvant immunotherapy in patients with resectable HCC are listed in Table 4 below [Refs. [44,60,64,65,66,67]]. Adjuvant immunotherapy for resectable HCC is intended to prevent postoperative recurrence; however, it remains an area of ongoing clinical investigation with evolving guidelines. Although early studies indicated promise, subsequent results have been inconsistent, and standardized protocols are still under development. Current evidence indicates that the greatest benefit from adjuvant immunotherapy is achieved by stratifying patients according to high-risk clinical features. Patients with microvascular invasion (MVI), substantial tumor burden, or multiple nodules are considered primary candidates for these therapies. Recent clinical research increasingly emphasizes the use of circulating tumor DNA (ctDNA) and tumor-infiltrating lymphocytes (TLs) to identify individuals with minimal residual disease who are most likely to benefit from adjuvant checkpoint inhibitors. Due to the variable outcomes observed in large, randomized trials, adjuvant systemic therapy, including immunotherapy, has not been universally adopted as the standard of care worldwide, and active surveillance remains the preferred approach for patients at lower risk. Table 5 provides an overview of all ongoing clinical trials investigating adjuvant and neoadjuvant ICIs in resectable and borderline-resectable HCC.

### 3.4. Immunotherapy Trials for Orthotopic Liver Transplantation-Treated HCC

About 30% of patients with HCC will come off the waiting list due to disease progression, even though liver transplantation (LT) provides a therapeutic alternative with a curative purpose. The 6-month waiting period before liver transplantation, fluctuating wait durations because of organ shortages, and unpredictable illness progression or mortality are the key variables linked to the dropout rate [68]. These figures demonstrate how important it is to start HCC targeted treatment before transplantation, including bridging therapy and downstaging therapy to treat tumors while waiting for organ allocation. Immunotherapy primarily plays a supplementary or alternative role in these settings, with transarterial and ablative therapies serving as the main tools for downstaging and bridging therapy. Immunotherapy, on the other hand, mostly plays a complementary or alternative role in these contexts. For bridging or downstaging regimens, small studies have examined immunotherapy as monotherapy, in combination with other immunotherapeutic drugs, and as a (neo)adjunct to resection, ablation, or transarterial treatments. Atezolizumab and bevacizumab have been shown in case reports to reduce tumor burden before transplantation, and pathological response rates of 20% have been reported in limited neoadjuvant trials [57]. To further understand the role of immunotherapy prior to liver transplantation, clinical trials are now underway [Table 6, Refs. [69,70,71,72]].

While neoadjuvant immunotherapy before liver transplantation for HCC has demonstrated encouraging outcomes in recent clinical studies, the statistical significance of these findings is constrained by small sample sizes and single-arm study designs. Future research should incorporate larger cohorts and well-structured control groups to properly evaluate the therapeutic potential of neoadjuvant immunotherapy in this context. Such improvements are crucial for guiding appropriate decision-making in HCC treatment. The scientific community is keenly anticipating the results of ongoing prospective clinical trials. Despite clear indications for treatment, the role of immunotherapy following transplantation has not been extensively explored. Recurrence of the disease occurs in 6% to 18% of patients undergoing liver transplantation for HCC [73]. Globally, the timing of immunotherapy post-transplantation is critical. Patients with a longer duration post-transplantation tend to have a reduced risk of rejection. As regards rejection mechanisms, three types can be distinguished. The first one is cell-mediated rejection, which is classified as either early, if it occurs within 90 days of transplantation, or late, if it occurs beyond 90 days. Early T-cell-mediated rejection is more common and generally occurs within the first month after transplantation [74]. Late T-cell-mediated rejection has unique histologic features (interface or lobular hepatitis, plasma cell infiltrates, and perivenulitis). Late T-cell-mediated rejection has been associated with higher rates of graft loss and worse OS compared with early T-cell-mediated rejection [75]. An atypical variant of T cell-mediated rejection is plasma cell-rich rejection, which occurs late (6 months after transplantation). Chronic rejection generally has a worse prognosis than acute T cell-mediated rejection due to its minimal response to steroids. Factors associated with chronic rejection include direct immunologic injury and indirect ischemic damage.

In contrast to T cell-mediated rejection, chronic rejection is associated with less inflammatory and more fibrotic and permanent injury. Antibody-mediated rejection (AMR) is another known mechanism of transplant rejection. Facets of AMR diagnosis include circulating donor-specific antibodies (DSA), C4D staining, and inflammation/fibrosis. AMR should be suspected in cases of nonadherence to immunosuppression [76]. The diagnosis of AMR is made based on a combination of a histopathological injury pattern consistent with acute AMR, positive serum DSAs, diffuse microvascular C4d deposition, and exclusion of other insults that might cause a similar pattern of graft injury. Careful timing of ICI administration before liver transplantation is critical to reduce the risk of allograft rejection. A recent multicenter retrospective cohort study found that maintaining at least a 30-day interval between the last ICI therapy and LT independently reduces the risk of allograft rejection.

A previous review highlighted the need for a minimum 30-day washout period to lower rejection risk. Although the optimal ICI washout period remains under debate, conducting an LT with an interval following ICIs of less than 30 days should be avoided, and intervals longer than 4 to 8 weeks are recommended [77]. On average, the interval between liver transplantation and the use of ICI was six weeks. Notably, research by Kayali et al. indicated that patients who responded to ICI therapy had a longer interval between liver transplantation and ICI treatment (six years compared to three years) than those who did not respond [78]. The optimal interval between ICI treatment and LT remains uncertain. The authors reported a median washout period of 94 days for patients with less than a 20% risk of allograft rejection. However, even with extended washout periods, up to 20% of patients may still experience rejection [79]. Individualized decisions regarding ICI cessation before LT should consider each patient’s risk factors for post-LT rejection and adverse outcomes. The authors also noted that adjusting the interval between the last ICI dose and LT is challenging because donor availability is unpredictable and affects washout timing [77]. A 42-day washout period before LT is generally considered the safest approach for patients with HCC receiving atezolizumab, nivolumab, or pembrolizumab. Some researchers propose that extending the washout period beyond three months may further reduce rejection rates [29]. Evidence indicates that neoadjuvant immunotherapy before LT is associated with favorable pathological responses, safety, and improved prognosis. Nevertheless, additional prospective studies and validated biomarkers are required to confirm the safety and efficacy of immunotherapy administered before and after LT. Ongoing clinical trials are addressing these questions (trial nos.NCT04425226 and NCT05027425).

We suggest that predictive models based on regional and temporal organ donation trends could help align ICI cessation with anticipated transplant timing. Incorporating such models into transplant planning may improve management of ICI washout periods.

The unusual immunological environment following transplantation entails careful evaluation of adjuvant immune cell therapy. The immunosuppression required to avoid graft rejection increases the risk of tumor recurrence. Studies have investigated the use of CAR T-cell immunotherapy targeting TAAs to overcome this problem, and the results have shown strong antitumor effects [80]. Following curative treatment, autologous CIK cell therapy has shown encouraging improvements in OS and RFS in HCC patients. The principal advantage of administering CIK cells more than once postoperatively is their ability to sustain an immune response. Based on this strategy, a phase I/II trial to assess the use of these methods as adjuvant therapy following LT for HCC is presently underway [80].

A multicenter study found that the absence of calcineurin inhibitor (CNI) use at ICI initiation is significantly associated with acute rejection in LT recipients with HCC. Rejection typically occurs early and is linked to high mortality. The use of ICIs in LT recipients remains controversial due to the risk of rejection. This study observed a 13% rejection rate, lower than previously reported rates of 28% to 39%, likely attributable to proactive optimization of immunosuppression (IS) in more than half of patients [81]. Although rejection now affects a smaller proportion of patients, ICI-induced cases tend to occur soon after treatment initiation, are challenging to manage, and are associated with high mortality. These findings highlight the potential for ICI use following LT, provided that treatment is approached with caution and individualization. Patients with earlier-stage HCC and preserved liver function might potentially benefit more from pre-transplant ICI therapy. Moreover, molecular profiling of tumors to identify biomarkers predictive of response to ICIs might help select candidates more likely to respond favorably to this treatment. Table 7 summarizes both completed and ongoing clinical trials on Adjuvant Immunotherapy for Liver Transplantation Among Patients with Hepatocellular Carcinoma.

## 4. Discussion

Surgery continues to be the primary treatment option for patients with resectable HCC. The therapeutic approach to HCC has the potential to undergo substantial transformation following the introduction of immunotherapy. Nonetheless, while notable progress has been made, further refinement of current therapeutic regimens, discovery of additional benefits from immunotherapy, and integration of these findings into treatment protocols are crucial for sustained advancement. Despite commendable efforts, no adjuvant treatment has been conclusively proven effective for early-stage HCC. The IMbrave 050 study was the first phase III study to demonstrate a benefit of adjuvant therapy in RFS. However, updated analyses presented at the European Society of Medical Oncology Congress 2024 demonstrated that the RFS benefit was not sustained after extended follow-up, while mature OS data remain pending. It is important to note that the adjuvant use of anti-angiogenic agents like bevacizumab has not been validated as an effective strategy. A recent meta-analysis indicated that studies involving ICI monotherapy or ICI-TKI combinatory adjuvant regimens consistently showed RFS benefits and a trend towards improved OS, suggesting that ICIs, with or without TKIs, might be more promising in this context [52]. The results of ongoing phase III trials, such as the EMERALD-2 trial and Keynote-937, along with long-term OS data, may ultimately influence clinical practice, as delayed effects and crossing hazards associated with ICIs could be observed. Tumor response rates of up to 30% can be achieved by combining targeted therapies with immune checkpoint inhibitors, offering promising options for neoadjuvant therapy. Analysis of the six completed trials (Table 3) evaluating adjuvant immunotherapy in resectable HCC at high risk for recurrence, each with a substantial patient cohort, revealed that two studies demonstrated improved RFS with immunotherapy. Additionally, one study reported a lower recurrence rate in the adjuvant immunotherapy group compared to the surveillance group. These findings indicate a potential benefit of adjuvant immunotherapy for high-risk patients, defined by factors such as presence of microvascular or macrovascular invasion, multinodular tumors, tumor size greater than 5 cm, poor differentiation, and elevated alpha-fetoprotein (aFP) or des-gamma-carboxy prothrombin (DCP) levels.

Administering neoadjuvant immunotherapy before surgical resection can lead to high rates of pathological response and prolonged survival without recurrence, in addition to enhancing anticancer immunity. Immunotherapy represents a novel systemic treatment for solid tumors. Monotherapy with ICIs targeting PD-1, such as nivolumab or pembrolizumab, initially showed moderate response rates and faces resistance mechanisms like the expansion of immunosuppressive cell populations and the activation of alternative immune checkpoint pathways. Current investigations into neoadjuvant therapy for resectable HCC involve patients who meet surgical resection criteria. Nevertheless, these criteria encompass a diverse group of patients with varying liver disease etiologies, liver function backgrounds, and tumor characteristics.

Additionally, ongoing research is essential to identify the most accurate biomarkers, such as PD-1/PD-L1, tumor mutation burden (TMB), circulating tumor DNA (ctDNA), microsatellite stability, DNA mismatch repair, neutrophil/lymphocyte ratio, cytokines, and cellular peripheral immune response, to optimize systemic treatment sequencing in HCC patients. Among the seven completed clinical trials on neoadjuvant immunotherapy for resectable HCC (Table 2) and the three completed trials for borderline-resectable HCC with curative intent (Table 3), most studies reported a high major pathological response (MPR) rate. Additionally, four studies reported satisfactory objective response rates. Although both the number of studies and enrolled patients remain limited, current evidence tentatively suggests that neoadjuvant therapy may significantly contribute to the treatment of patients with borderline-resectable HCC and those at high risk of recurrence.

However, as mentioned above, most studies on neoadjuvant therapy involve limited patient populations, and their findings necessitate long-term evaluation in rigorously designed clinical trials to clarify the implications of neoadjuvant treatment for HCC resection, particularly at locally advanced and advanced stages.

In the context of a comparative analysis between neoadjuvant and adjuvant immunotherapy, the identification of effective perioperative or adjuvant treatments remains a critical unmet need for patients with HCC, particularly given the high incidence of early postoperative relapse. Neoadjuvant therapy is increasingly recognized as an integral component of perioperative management, as anti-PD(L)1 antibodies possess a half-life exceeding three weeks and persist in circulation following surgical intervention. Neoadjuvant ICIs can also downstage tumors, thereby enhancing surgical outcomes. Moreover, neoadjuvant ICIs may induce a more robust immune response than adjuvant ICIs, largely due to the systemic immunosuppressive effects of invasive surgery. A comparative study that analyzed 1373 relevant references demonstrated that comprehensive quantitative comparisons between neoadjuvant and adjuvant immunotherapy in perioperative cancer patients have revealed a significant shift in research focus [84]. Since 2020, neoadjuvant immunotherapy has become more prominent than adjuvant immunotherapy [84]. Recent reviews further indicate a clear transition toward neoadjuvant and perioperative clinical trials, as well as the development of personalized neoantigen vaccines [85]. Adjuvant ICIs typically activate antitumor immunity against micrometastases after removal of the primary tumor. In contrast, neoadjuvant immunotherapies utilize the primary tumor burden as an antigen source to initiate the immune response. Previous studies have demonstrated that resection of HCC disrupts the tumor immune barrier, leading to reduced infiltration of effector immune cells, such as CD8+ T cells, and decreased efficacy of adjuvant therapy. In contrast, neoadjuvant therapy does not appear to impair immune cell infiltration [86]. Further research indicates that neoadjuvant PD-1 inhibitors enhance antitumor immunity by promoting the induction and maturation of tertiary lymphoid structures (TLS), which may facilitate the elimination of residual postoperative micrometastases [87]. HCC tumors containing TLS show significantly increased intratumoral infiltration of CD3+CD8+ T cells and B cells, and patients with these tumors experience significantly prolonged RFS following adjuvant PD-1 inhibitor therapy. The extended RFS (42.1 months) observed with the perioperative strategy in the CARES-009 trial may be closely associated with TLS induction by neoadjuvant treatment [44]. In contrast, the IMbrave050 trial did not include TLS-related detection, potentially overlooking the optimal target population.

Another important consideration is the use of immune checkpoint inhibitors as monotherapy or in combination therapies for HCC. Clinical trials investigating ICI monotherapy in HCC have shown both efficacy and tolerability. However, these studies have not demonstrated clear superiority over sorafenib, prompting exploration of combination strategies to enhance therapeutic outcomes. ICI combinations, typically comprising an anti-PD-L1 or anti-PD-1 agent with an anti-CTLA-4 agent, have demonstrated improved response rates compared to monotherapy, but are also associated with increased rates of adverse events [88]. Multiple clinical trials, such as the phase II trial NCT02519348 and HIMALAYA (a phase III trial), have evaluated the efficacy and safety of anti-CTLA-4 and anti-PD-L1 combination regimens in advanced HCC. The Checkmate 040 trial demonstrated promising efficacy and tolerability of ICI combinations for advanced HCC. Based on these findings, the combination of nivolumab and ipilimumab received FDA approval for advanced HCC, establishing it as the standard of care in the second-line setting for patients who have progressed on prior TKI therapy, have no contraindications to ICIs, and can tolerate the increased toxicity associated with dual ICI therapy.

In contrast, ICI monotherapy with pembrolizumab (or off-license nivolumab) in the second-line setting is reserved for patients with reduced performance status [88].Selection of an immunotherapy regimen requires consideration of multiple factors, including tumor burden, liver function status (for example, Child–Pugh score), prior treatment history, and relevant biomarkers. In the first-line setting, combination immunotherapy is generally preferred. The IMbrave150 trial demonstrated the efficacy of Atezolizumab plus Bevacizumab in this context. For patients with disease progression after first-line immunotherapy, Nivolumab plus Ipilimumab has demonstrated an objective response rate of 32%, as reported in the CheckMate 040 trial. The HIMALAYA trial has also provided evidence supporting Tremelimumab plus Durvalumab as a second-line option for patients whose disease progresses following Atezolizumab and Bevacizumab treatment. Monotherapy with ICIs, such as Pembrolizumab, is a potential option for patients who cannot tolerate dual immunotherapy. Evidence from the KEYNOTE-240 trial supports its efficacy as a second-line treatment [89].

Safety concerns associated with systemic therapies in the neoadjuvant setting are justified due to the risk of severe side effects that may delay or preclude curative surgery. Therefore, these risks and their influence on event-free survival require thorough evaluation in clinical trials. Additionally, collecting matched tissue samples at both biopsy and resection offers a significant opportunity for translational research in early-stage hepatocellular carcinoma (HCC) systemic therapy studies.

A prominent area of current discussion concerns the comparison between transplant and non-transplant settings. Surgical therapies, particularly LT, are associated with excellent long-term survival in patients with HCC, particularly compared with non-surgical treatments. The criteria for LT are widely accepted and continue to evolve, primarily focusing on selecting patients with less aggressive HCC. These criteria are designed to identify HCC patients with the potential for long-term survival following transplantation and to exclude those unlikely to benefit. Studies report that 30.4% of the total cohort ultimately undergo LT, and their survival rates are significantly higher than those of patients receiving liver resection, systemic therapy, or active surveillance [90]. Liver transplant recipients typically exhibit poorer liver function than non-transplant patients but present with less aggressive HCC, characterized by smaller tumors, lower serum alpha-fetoprotein (AFP) levels, and a lower incidence of PVT [90].

In contrast, the non-transplant group often includes patients with larger tumors, elevated serum AFP levels, and macroscopic PVT. The reasons many patients do not qualify for transplantation require further investigation. Given the substantial survival differences between patients treated with LT and those who are not eligible, it is likely that factors beyond treatment selection, such as tumor biology, contribute to these outcomes [91].

## 5. Conclusions

Clinical and pathological considerations of recurrence risk guide the decision to apply these agents or not. Both ICIs and immune-cell therapies have demonstrated early encouraging results in reducing recurrence and boosting survival among patients undergoing liver resection or LT. However, most neoadjuvant, perioperative, and adjuvant ICI strategies remain investigational, and despite promising results, a clear survival benefit has not yet been established. Moreover, challenges such as the low immunogenicity of HCC, the immunosuppressive tumor microenvironment, and the need to balance graft tolerance with antitumor immunity in LT recipients persist. Despite advances in biomarker development to predict response to ICIs, no biomarker exists to identify patients with HCC most likely to benefit from these therapies. Future trials testing ICIs in the (neo)adjuvant setting should adhere to these principles to enable more accurate application of this essential therapeutic class in early-stage HCC. Numerous phase III trials are exploring combinations of TKIs (Lenvatinib, cabozantinib, and apatinib) and ICIs, as well as combinations of CTLA4 inhibitors (ipilimumab and tremelimumab) and other ICIs.

Although initial results of neoadjuvant immunotherapy are encouraging, they should be interpreted with caution, given the limited sample sizes in existing studies, the potential for bias, and the lack of long-term confirmation.

Overall, there is growing scientific and clinical evidence that immunotherapy will play a central role, alongside surgery, in successful management with curative intent of early, intermediate and advanced stage hepatocellular carcinoma in the near future. This has already been proven for advanced-stage HCC as sole medical management. The current evidence partially supports this assumption. Ongoing and future large-scale randomized studies will soon clarify the exact role of immunotherapy in the management of HCC.

## 6. Future Directions

Despite the benefits in progression-free and overall survival offered by checkpoint inhibitors, the proportion of patients achieving long-term disease control remains limited. Therefore, biomarker stratification is vital to prevent overtreatment. Liquid biopsy-based screening tests have gained interest for early diagnosis in asymptomatic individuals. Detection of tumor-derived circulating RNA and innovative protein-based technologies are anticipated to yield clinically valuable biomarkers. Common biomarkers include PD-L1, TMB, and pathological response following neoadjuvant chemoimmunotherapy, which can be broadly categorized as tumor cell-intrinsic and microenvironment-related.

HCC typically arises from chronic liver inflammation, leading to the formation of a complex tumor microenvironment (TME) comprising immune and stromal cells. Characterizing the TME in HCC is challenging, yet it plays a critical role in metastasis and tumor resistance. The complex interactions among immune, stromal, and cancer cells within the TME have driven the development of immunotherapies that specifically target this microenvironment. The regulatory roles of immune checkpoint ligands and receptors in modulating immune cell activity justify the use of ICIs in HCC. Cancer cells utilize various immune escape mechanisms that affect immunotherapy outcomes, including increased PD-L1 expression, altered antigen presentation, and loss of interferon gamma (IFNg) sensitivity [92]. Immune cells express ligand–receptor immune checkpoints that can either stimulate or inhibit their function. Stimulatory immune checkpoints include CD28, GITR, and OX40, while inhibitory checkpoints comprise PD-1, CTLA4, LAG3, TIGIT, and TIM3 [35]. The principal clinical applications of ctDNA analysis include monitoring therapeutic response, detecting minimal residual disease (MRD) following treatment, and facilitating cancer detection. Patients with advanced malignancies typically exhibit the highest average ctDNA concentrations [93].

In contrast, during MRD screening for early-stage tumors, ctDNA levels may fall below one part per million, necessitating the use of highly sensitive assays. Literature across various cancer types indicates that patients with undetectable ctDNA following treatment experience significantly improved prognoses compared to those who remain ctDNA-positive. Biomarkers such as ctDNA are expected to transform cancer therapy by enabling the identification of genetic mutations and supporting personalized treatment strategies that enhance efficacy and minimize adverse effects. The integration of ctDNA into therapeutic protocols represents a paradigm shift, wherein biomarkers serve not only diagnostic purposes but also facilitate ongoing disease monitoring. Predictive biomarkers are critical for optimizing treatment regimens and ensuring that therapies are both individualized and effective. However, data on biomarkers to guide therapy for advanced HCC remain limited. A meta-analysis involving more than 3500 patients demonstrated that tumor PD-L1 expression correlates with poorer prognosis in HCC, including associations with poorly differentiated tumors, elevated α-fetoprotein levels, and reduced OS. Non-randomized studies of nivolumab and pembrolizumab have reported higher rates of radiographic response and prolonged progression-free survival (PFS) in patients exhibiting positive tumor PD-L1 expression or combined tumor and non-tumor PD-L1 expression scores. Last, LAG-3 is an emerging immune checkpoint target and functions as an inhibitory receptor that regulates T cell activation. Its expression is upregulated in tumor-infiltrating CD8+ T cells across various tumor types, including HCC. In a study of tumor samples from resected HCC patients, LAG-3 expression on tumor-infiltrating lymphocytes (TILs) was observed in 65% of samples, and PD-L1 expression in 83%. Multiple clinical trials are currently evaluating combinations of LAG-3 inhibitors with anti-PD-1 therapy in advanced HCC [94].

## Figures and Tables

**Table 1:** Compares Western and Asian guidelines for HCC management.

Type of Therapy	ASCO	AASLD	EASL	ESMO	Asian Guidelines (CSCO/APASL/KLCA-NCC)
Neoadjuvant therapy	Not recommended outside clinical trials; insufficient evidence for routine use	Not recommended; prioritize upfront resection or transplantation in eligible patients	not recommended outside trials due to lack of evidence and risk of delaying curative therapy	not recommended outside trials; surgery remains standard for resectable disease	CSCO recommends neoadjuvant TACE or HAIC ± ICIs in selected patients (e.g., borderline resectable); APASL/KLCA-NCC allow individualized approaches
Adjuvant therapy	Recommends consideration of adjuvant atezolizumab + bevacizumab in high-risk patients (based on IMbrave050), pending OS confirmation	Similar conditional recommendation; emphasizes evolving evidence and need for longer follow-up	not recommend routine adjuvant systemic therapy; requires robust phase III OS benefit	not recommend routine adjuvant therapy; suggests surveillance or clinical trial enrollment	APASL: insufficient evidence for routine use; KLCA-NCC: allows adjuvant immunotherapy (e.g., CIK cells) based on regional trials; CSCO: more permissive in high-risk patients
Downstaging	Recognized primarily to enable liver transplantation (e.g., within Milan criteria)	Endorsed for transplant eligibility using locoregional therapies	Recommended for selected patients to achieve transplant candidacy	Recognized mainly for transplant selection	Broader application; actively used to expand both transplant and resection eligibility
Conversion therapy	Not established as a standard therapeutic strategy	Not explicitly recommended as a treatment pathway	Not recommended as standard; limited evidence for routine use	Not considered a standard strategy	Actively pursued in intermediate/advanced disease; combination regimens (TKIs + ICIs ± HAIC) used to achieve resectability in selected patients
ICI + locoregional combinations	Promising but investigational; recommended within clinical trials	Acknowledged but not standard; requires high-level evidence	Recognized rationale but not standard of care	Supported conceptually but limited to trials/selected cases	More permissive; increasingly incorporated into practice based on regional evidence, though still evolving

Abbreviations: ASCO, American Society of Clinical Oncology; AASLD, American Association for the Study of Liver Diseases; EASL, European Association for the Study of the Liver; ESMO, European Society for Medical Oncology; CSCO, Chinese Society of Clinical Oncology; APASL, Asian Pacific Association for the Study of the Liver; KLCA-NCC, Korean Liver Cancer Association–National Cancer Center; TACE, transarterial chemoembolization; HAIC, hepatic arterial infusion chemotherapy, ICIs, Immune Checkpoint Inhibitors, TKIs, Tyrosine Kinase Inhibitors.

**Table 2:** Completed clinical trials regarding neoadjuvant immunotherapy in resectable HCC.

Study Design	Phase	Patients	Reference/ Authors	Safety	Standardized Comparator	Primary Endpoint and Key Efficacy Result	Publication Status
combination therapy with cabozantinib and nivolumab prior to surgery	Ib	15	NCT03299946/ Won Jin Ho et al. [42]	manageable safety profile	not a standardized comparator	R0 resection 80% 42% MPR (5 pts in 12 underwent surgery)	Published 2021
1st arm: nivolumab alone before and after surgery 2nd arm: nivolumab plus ipilimumab before and after surgery.	II	30	NCT03222076/ Ahmed Omar Kaseb EH et al. [43]	Nivolumab: 23% gr 3 AE, Nivolumab + Ipilimumab: 43% grade 3–4 AEs	nivolumab monotherapy	Safety and tolerance 33%MPR, 23% ORR	Published 2022
Drug: Durvalumab 1500 mg IV + Tremelimumab 300 mg IV, single infusion about 2 weeks after first DEB-TACE procedure. Drug: Durvalumab (monotherapy) 1500 mg IV infusion every 4 weeks, for maximum 13 doses/cycles.	II	21	NCT03638141/ Amy K. Kim et al. [46]	20% gr 3 or higher AEs	not a standardized comparator	ORR 55% PFS 6.1 OS 28.8 mo	Published 2025
1st arm: a single dose of Toripalimab before surgery. 2nd arm: Toripalimab plus daily oral Lenvatinib prior to surgery.	II	40	NCT03867370/ Ying-Hong Shi et al. [47]	no unexpected toxicities	Group C Participants receive a single dose of Toripalimab with envatinib. Post-operative Phase: Toripalimab every 3 weeks for up to 48 weeks.	20% MPR no pathological complete response	Published 2021
1st arm: Patients with a partial response or reduced stable disease (PR/SD) proceed to surgery followed by adjuvant tislelizumab. 2nd arm: Patients with progressive disease (PD) receive 2 cycles of combined tislelizumab and envatinib, followed by surgery and adjuvant treatment	II	50	NCT05807776/ Ruyu Han et al. [48]	11.1% gr 3 AEs	not a standardized comparator	30.6% ORR (mRECIST) 33%MPR 15.3 pCR	Published 2025
Camrelizumab With Apatinib for Perioperative Treatment	II	31	NCT04701060/ Yunlong Cui et al., [49]	Tolerable safety profile 22.6% gr4 AEs	not a standardized comparator	MPR 38.5% ORR 13.8% based on RECIST 1.1 and 48.3 based on mRECIST criteria.	Presented at the 2023 ASCO Annual Meeting on 31 May 2023
IMMULAB Pembrolizumab on day 1 of cycle 1 and 2 RFA/MWA or TACE with RFA, MWA or brachytherapy on day 1 of cycle 3 Pembrolizumab 2 after local ablation Pembrolizumab for 12 months	II	30	NCT03753659 Arndt Vogel, et al. [45]	well tolerated	not a standardized comparator	Objective response rate (ORR) according to RECIST 1.1. ORR was 13.3%, with 6.7% complete responses (CR) and 6.7% partial responses (PR) after two cycles. 2 years OS	Published 2025

**Table 3:** Completed clinical trials regarding neoadjuvant immunotherapy in borderline-resectable HCC with curative purpose.

Name and Study Design	Phase	Patients	Reference/ Authors	Safety	Standardized Comparator	Primary EP and Key Efficacy Result	Publication Status
stage A/B HCC beyond Milan criteria CAM, APA and mFOLFOX7. Surgical feasibility was evaluated every 2 cycles. Sequential therapy 2 weeks after surgery. CAM and APA up to 12 months.	II	12	NCT06670107 /Linhui Peng et al. [50]	Grade 3–4 adverse events in approx. 8.3%, suggesting the combination is generally manageable.	not standardized comparator	Improvement R0 rates 11 pts have undergone hepatic surgery, R0 resection 100%, (7/11) 63.6% MPR.	Published 2025
Nivolumab plus ipilimumab on day 1 of each cycle (every 3 weeks). Tumor assessment after 6 weeks and 12 weeks.	II	43	NCT03510871/Yih-Jyh Lin et al. [55]	manageable toxicity profile	not standardized comparator	>10% decrease of the sum of the target lesions according to RECIST 1.1 MPR 18.6% pCR 25% OS 60%	Published 2025
Nivolumab for 3 doses → Hepatectomy 2 weeks after the 3rd dose of nivolumab	II	20	NCT05471674 /Yajing Zhang et al. [56]	Surgical feasibility, no surgical delays. manageable safety profile	A standardized anti-PD-1 response score calculated from cfDNA as a genomic comparator to predict therapeutic efficacy.	MPR. MPR 36.8% Surgical feasibility 95%, Complete response 15%	Published 2023 and 2025

**Table 4:** Completed clinical trials evaluating adjuvant immunotherapy in patients with resectable HCC at high risk for recurrence.

Study Design	Phase	Patients	Reference/ Authors	Safety	Standardized Comparator	Primary EP and Key Efficacy Result	Publication Status
SILVER Randomized 1st arm: rapamycin inhibitor-free immunosuppression 2nd arm: incorporating sirolimus	III	525	NCT00355862 /Edward K Geissler et al. [64]	serious AEs were nearly identical, with 874 events reported in the sirolimus cohort/860 in the control group	mTOR-inhibitor-free immunosuppressive protocol.	RFS (64.5% in 1st arm, 70.2% in 2nd arm) OS	Published 2016
1st arm: adjuvant immunotherapy (autologous CIK cells) 2nd arm: no therapy	III	230	NCT00699816 /Joon Hyeok Lee et al. [60]	7.8% in the immunotherapy group vs. 3.5% in the control group	no treatment-control group	1st arm RFS 44 months 2nd arm RFS 30 months Risk of death due to liver cancer was significantly reduced (HR: 0.19)	Published 2015
1st arm: sintilimab in resected high-risk HCC. 2nd arm: active surveillance	II	198	ChiCTR2000037655 /Kang Wang et al. [65]	Gr 3 or 4 AEs in 12.4% in the 1st arm	active surveillance	1st arm: 27.7 mo RFS 2nd arm: 15.5 mo RFS reduce risk of cancer recurrence by 46.6%	Published 2024
1st arm Tislelizumab combined with TKI 2nd arm; active surveillance	II	60	NCT06059885 /Shida Pan et al. [66]	4.1% gr 4 AEs	active surveillance	Recurrence rate (RR) 20.83% in 1st arm RR 58.3 active surveillance Improvement in DFS	Published 2024
CARES-009 1st arm: Preoperative camrelizumab with apatinib for patients with resectable HCC at high risk of recurrence 2nd arm: radical surgery alone	II/III	294	NCT04521153 Zheng Wang et al. [44]	38% grade 3 or worse AEs	Surgery alone patients	1. Event-free survival 2. MPR EFS 42.1 months in the 1st arm vs. 19.4 months with surgery alone. MPR 35% in the 1st arm vs. 8% in the 2nd	Published 2025
IMbrave050 Atezolizumab plus bevacizumab	III	668	NCT04102098 Adam Yopp et al. [59]	AEs were consistent with the known toxicity profiles of each individual drug	active surveillance	RFS OS data remain immature as median OS has not been reached	Updated Follow-Up Data (2026)
KEYNOTE-937 Ist arm: pembrolizumab 2nd arm: placebo	III	959	NCT03867084 Stephen Chan et al. [67]	Grade 3 or 4 Es occurred in 14% in the pembrolizumab arm compared to 5% in the placebo arm	placebo	RFS, OS No difference between the two arms	Published 2026

**Table 5:** Ongoing clinical trials of adjuvant and neoadjuvant ICIs in resectable and borderline-resectable HCC.

Name and Study Design	Phase	Patients	Reference	Efficacy	Standardized Comparator	Safety	Primary EP	Publication Status
neoadjuvant cemiplimab in patients with resectable HCC → surgical resection → additional eight cycles of cemiplimab (2019–2030)	IIa	65	NCT03916627	Perioperative cemiplimab with or without SBRT for resectable HCC.	no data available (NA)	Well tolerated	Tumor necrosis (>70% necrosis of the resected tumor).	Published peer-reviewed papers and conference presentations
TALENT. Tislelizumab with or without Lenvatinib (2020–2027)	II	80	NCT04615143	Neoadjuvant tislelizumab alone or combined with lenvatinib resectable recurrent HCC	No comparator	Strong baseline safety	1-year DFS	Results pending publication
PRIMER-1 Pembrolizumab or Lenvatinib or combination (2022–2030)	II	60	NCT05185739	perioperative pembrolizumab, lenvatinib, or their combination in resectable HCC	arm 1: Monotherapy with Pembrolizumab. arm 2: monotherapy with Lenvatinib. experimental arm: Combinationtherapy of Pembrolizumab + Lenvatinib.	Toxicity analogous to that of the individual drug.	MPR	Results pending publication
MORPHEUS 1st arm: atezolizumab + bevacizumab until surgery or toxicity. 2nd arm: atezo+ bev+ tiragolumab until surgery or toxicity. 3rd arm: Tobemstomig + Bev until surgery or toxicity. 2023–2028	Ib/II	150	NCT05908786	pen-neoadjuvant, pre-surgical ICI combinations. (a) Atezolizumab + Bevacizumab (b) Atezolizumab + Bevacizumab + Tiragolumab (c) Tobemstomig + Bevacizuma	atezolizumab plus bevacizumab	Grade 3–4 AEs 11–27%. 0% unexpected delays to surgery due to toxicity.	MPR	no peer-reviewed publications
1st arm: Ipilimumab +pembrolizumab or (Ipi) +durvalumab 2nd arm: (Ipi) +(pembro) or (Ipi) +(durva) with idarubicin 3rd arm: (Ipi) +(pembro) or (Ipi) +(durva) with (ida) plus bevacizumab (2024–2033)	II	90	NCT06492408	No data available	no standardized comparator	No data available	MPR	pending published results
NEOTOMA 1 dose Tremelimumab with Durvalumab at cycle 1 and 1 cycle of Durvalumab pre surgical resection. Adjuvant Durvalumab (2023–2026)	II	28	NCT05440864	Durvalumab and Tremelimumab as a neoadjuvant treatment for patients with resectable HCC.	no standardized comparator	Accepted safety profile	According to RECIST v1.1 and mRECIST Objective response rate ORR AEs	Pending published results
Tislelizumab With Sitravatinib as Adjuvant Therapy in HCC at High Risk of Recurrence After Curative Resection (2022–2026)	II	40	NCT05407519	evaluating the combination of tislelizumab plus sitravatinib as adjuvant therapy for HCCat high risk of recurrence	no standardized comparator	40.9% Grade ≥ 3 AEs	2-year RFS	Preliminary data (2025 ASCO Annual Meeting Abstract).
KEYNOTE-937 1st arm: IV pembrolizumab as adjuvant therapy 2nd arm: placebo (2019–2029)	III	950	NCT03867084	Pembrolizumab as adjuvant therapy to prevent recurrence in HCC after surgical resection or local ablation	IV placebo administered every 3 weeks.	14% Grade ≥ 3 AEs	RFS (46.7 months with pembrolizumab, 45.5 months with placebo. OS	Primary publication ASCO Publications 2026
HESTIA Tislelizumab until progression or toxicities, for a max duration of 2 years (2023–2028)	II	50	NCT05622071	no data available	no standardized cmparator	no signal of increased toxicity	ORR	Preliminary data in a peer-reviewed medical journal.
Adjuvant treatment with Donafenib: Treatment cycle Envafolimab: Treatment cycle Donafenib and Envafolimab until recurrence, toxicity, death, withdrawal of consent or completion of 16 cycles. (2024–2027)	II	45	NCT06498622	no data available	no standardized comparator	no data available	RFS OS	Pending published results
Adjuvant atezolizumab and bevacizumab → surgery (2021–2027)	II	16	NCT04721132	Safety and efficacy of atezolizumab and Bevacizumab as a neoadjuvant treatment for resectable HCC	not standardized comparator	Toxicity analogous to that of the individual drug.	Pathologic complete response (pCR)	no published results
Regorafenib and durvalumab. every 28 days for 2 years from registration or until surgery, disease progression, toxicity, or patient withdrawal. (2023–2028)	II	30	NCT05194293	not yet published	no standardized comparator	no data available	ORR	Pending results
DUMELEP. Durvalumab/Tremelimumab infusion in a neoadjuvant setting followed by PA procedure Durvalumab infusions 2024–2028	II	30	NCT06045975	not yet published	no standardized comparator	no data available	RFS	no published results
CheckMate 9DX 1st arm: nivolumab 2nd arm: placebo (2018–2031)	III	545	NCT03383458	efficacy of adjuvant nivolumab versus a placebo in HCC at high risk of recurrence after curative resection or local ablation	a placebo	Grade I-II	RFS	no published results

**Table 6:** Current trials on neoadjuvant immunotherapy for HCC before liver transplantation.

Study Design	Phase	Patients	Primary EP and Key Efficacy Result	Reference/ Authors	Standardized Comparator	Safety	Publication Status
COMPLETED TRIALS							
1st arm; ICI group (anti-PD-1; anti-PD-L1; anti-CTLA-4. 2nd arm: non-ICI group	Observational	160	Graft rejection 1st arm: Higher Rejection & Mortality Risk The Importance of Timing: A shorter duration between the last dose of immunotherapy and LT was linked to acute allograft rejection.	NCT05913583 /Li Pang et al. [69]	non-ICI exposure control group	Pretransplant immunotherapy increases the risk of rejection. Washout period of ≤21 days was linked to allograft rejection linked to high allograft rejection rate	Published 2025
PLENTY 202001 1st arm: Pembrolizumab with Lenvatinib. 2nd arm: active surveillance.	Randomized open-label	192	RFS 1st arm: 37.5% 2nd arm: 12.5%	NCT04425226 /Zicheng Lv et al. [70]	standard-of-care waitlisting practice	manageable safety profile	Published 2024
ImmunoXXL Atezolizumab and bevacizumab → transplantation. Efficacy and safety of LT for HCC in patients who are initially beyond standard transplant criteria.	Observational.	12	RFS 2-y OS 94% 2-y RFS 90% MPR 62.5%	NCT05879328 /Sherrie Bhoori et al. [71]	atezolizumab and bevacizumab maintained treatment alone	manageable, though elevated, risk of acute graft rejection.	Published 05/2026
DULECT 2020-1 locally advanced HCC before LT and patients with metastatic, unresectable HCC. Combination treatment durvalumab (1500 mg on Day 1 of each 28-day cycle) and oral lenvatinib	Single arm, open-label	20	RFS PFS Reducing the localized tumor burden	NCT04443322 /Hao Feng et al. [72]	not standardized comparator	accepted safety profile	Published 2021
REGISTRY ONLY STUDIES							
InterventionaL Experimental: HBV/TCR-T cell infusion.	I	13	Safety of the TCR-T treatment	NCT02686372	not standardized comparator	Met safety endpoints	Not yet formally published
SHR-1210. Open label. Camrelizumab plus apatinib. tests this combination therapy as a neoadjuvant “downstaging” or “bridging” treatment for patients with HCC waiting to meet the criteria for a curative LT	I/II	120	ORR, RFS.	NCT04035876	not standardized comparator	no data available	Not yet formally published
Comparison of two groups: 1st arm: HCC patients who received pre-transplant PD-1/PD-L1 inhibitors as bridging or downstaging therapy.2nd arm: control group: Matched HCC patients who underwent LTw ithout prior immunotherapy exposure.	Prospective, observational, case–control	60	Incidence and severity of allograft rejection. OS, RFS	NCT05411926	patients with HCC without pre-transplant PD-1/PD-L1 inhibitor monotherapy.	no data available	Not yet formally published
	ONGOING TRIALS						
evaluating the combination of atezolizumab and bevacizumab alongside TACE as a neoadjuvant/downstaging bridging therapy for LT.	IV	24	Acute allograft rejection rate	NCT05185505	not standardized comparator	-	Pending ongoing trials
combination immunotherapy, targeted therapy, and Hepatic Arterial Infusion Chemotherapy (HAIC) as a neoadjuvant strategy to downstage HCC prior to LT. Anti-PD1 with locoregional therapy.	II	59	Free survival	NCT05475613	not standardized comparator	-	Pending ongoing trials
ESR2021010 Single arm: Durvalumab with Tremelimumab in patients who are officially listed to receive a LT.	II	30	Acute allograft rejection rate	NCT05027425	not standardized comparator	-	Pending ongoing trials

**Table 7:** Summary of Clinical Trials on Adjuvant Immunotherapy for Liver Transplantation Among Patients with Hepatocellular Carcinoma [Refs. [82,83]].

Study Design	Phase	Patients	Primary EP and Key Efficacy Result	Reference /Authors	Standardized Comparator	Safety	Efficacy	Publication Status
COMPLETED TRIALS								
1st arm: LT + Adenovirus-mediated Adjuvant Therapy 2nd arm: LT	II	81	Improved OS and RFS	NCT02202564 /Ruidong Zhu et al. [82]	LT alone	well-tolerated	RFS 1st arm: 55.9% 2nd arm 13.5%, OS 60.3% 1st arm 2nd arm 19.3%	Primary results published 2018
intratumoral or adjuvant injection of ADV-TK (Adenovirus Thymidine Kinase) combined with LT in intermediate- or advanced-stage HCC.	II	40	OS	NCT00300521 /Mubalake Abudoureyimu et al. [83]	LT alone	highly favorable safety and tolerability profile	-3-year OS ~69.6%, compared to 19.9% in the control group.	Published 2019
ONGOING TRIALS								
1st arm: LT-only 2nd arm: LT+ADV-TK	III	180	PFS	NCT03313596	liver transplantation alone	no data available	No data available	Results not published yet
Dorafenib + TACE	II	20	RFS, safety	NCT05576909	no standardized comparator	not published	not published	Results not published yet
1st arm: Drug: (sorafenib/lenvatinib/regorafenib) bevacizumab 2nd arm: no intervention	II	88	1-year RFS OS safety	NCT06041490	Observa tion/without adjuvant therapy	Results not published yet	Results not published yet	Results not published yet
FOLFOX plus Lenvatinib	I-II	62	2-year RFS, safety	NCT06409637	no standardized comparator	Results not published yet	Results not published yet	Results not published yet

## Data Availability

This is a narrative review with a structured literature search. Major databases, including PubMed, EMBASE, Cochrane Library, and Scopus, were systematically searched for published studies. No new data were created.
